# Application of Food Waste in Biodegradable Composites: An Ecological Alternative in Tribology

**DOI:** 10.3390/ma18143216

**Published:** 2025-07-08

**Authors:** Łukasz Wojciechowski, Zuzanna Sydow, Karol Bula, Tomasz Runka

**Affiliations:** 1 Institute of Machines and Motor Vehicles, Poznań University of Technology, 60-965 Poznań, Poland; 2 Institute of Material Technology, Poznań University of Technology, 60-965 Poznań, Poland; karol.bula@put.poznan.pl; 3 Institute of Materials Research and Quantum Engineering, Poznań University of Technology, 60-965 Poznań, Poland; tomasz.runka@put.poznan.pl

**Keywords:** biodegradable composites, food waste, tribological materials, coefficient of friction, abrasive and adhesive wear, self-lubrication

## Abstract

Biodegradable composite materials enhanced with food waste for tribological applications are proposed in this article. Polymer materials used as matrices included polypropylene and polylactic acid, which, according to the manufacturers’ claims, were made entirely or partially from biodegradable raw materials. Additionally, the matrices were enhanced with three types of waste materials: powders derived from cherry and plum stones, and pomace extracted from flax seeds. The composites differed in the percentage content of filler (15 or 25 wt.%) and particle size (d < 400 µm or d > 400 µm). Thirty-minute block-on-ring friction tests were performed to determine frictional behaviour (when pairing with steel), and the wear mechanisms were analysed using optical microscopy and scanning electron microscopy, supplemented with Raman spectroscopy. A notable effect of cherry and plum stone fillers was observed as a reduction in motion resistance, as measured by the friction coefficient. This reduction was evident across all material configurations in polypropylene-based composites and was significant at the lowest concentrations and granulation in polylactic acid composites. The effect of flaxseed pomace filler was ambiguous for both composite bases.

## 1. Introduction

In recent years, there has been a dynamic improvement in materials used for parts in friction pairs, driven by sustainable development challenges and the increasing demands of industrial applications. While traditional materials like cast iron, steel, and copper alloys continue to dominate the market, these are increasingly being replaced by alternative materials that provide comparable durability and reliability while reducing negative environmental impacts. Composite materials with a polymer matrix including a filler with specific required properties warrant special attention in this aspect [1]. When designing composites, specific components can be selected to tailor the resulting material for particular applications, such as enhancing mechanical properties or reducing resistance to movement in friction pairs. Innovative approaches to creating tribological composites focus on both the creation of new materials (innovative combinations of matrices with filler/fillers) and new manufacturing techniques that offer better functional properties of the final product. Examples of the latest generation of tribological materials are composites in which the fillers are nanomaterials such as carbon nanotubes, graphene, nanoclays (e.g., montmorillonite nanoclay), or metal oxide nanoparticles (e.g., Al_2_O_3_, ZnO, and CuO) [2]. Another notable trend in tribological composites is the use of self-lubricating materials. This effect is achieved by incorporating suitable components, such as graphite, graphene, or molybdenum disulphide, which provide lubrication through the frictional release of substances during contact. In such cases, the material matrix is designed with dedicated pores that serve as reservoirs for solid lubricants, ensuring continuous and stable lubrication as these lubricants are gradually depleted due to friction [3].

While various types of matrices (metal, ceramic, or polymer) can be used in composites for tribological applications, polymer composites, due to their competitive properties, seem to have the most promise. However, it should be remembered that these, particularly those made from conventional polymers derived from petroleum processing, still pose significant environmental challenges. The production of these materials results in substantial carbon dioxide emissions, which contribute to an increased carbon footprint [4]. Additionally, traditional polymer composites have limited biodegradability, leading to the long-term accumulation of waste in the environment. This waste can break down into microplastics, which pose serious threats to both aquatic and terrestrial ecosystems [5]. Taking into account the above problems, it is essential to investigate alternative, more sustainable materials and develop technologies that improve recycling or biodegradation to minimise the negative impact on the environment. An approach that can solve this problem may be to use an appropriate configuration of matrix and filler materials. Considering the matrix, it is possible to use polymers made from renewable, biodegradable raw materials, which contributes to reducing the carbon footprint and facilitates the biodegradation process after the end of the product’s life cycle. Examples of this type of polymer include polypropylene (PP) made from 100% biocomponents or polylactic acid (PLA). Compared with traditional plastics, PLA biodegrades under industrial composting conditions, transforming into carbon dioxide and water in less than 90 days. Thanks to this, products made of PLA, after appropriate processing, do not pollute the environment with microplastics. It is worth noting, however, that the PLA biodegradation process requires specific conditions, such as appropriate temperature and humidity, which are available mainly in industrial composting plants. Therefore, it is crucial to ensure the proper management of PLA waste to maximise its environmental benefits [6]. As for PP, it is believed that its decomposition under atmospheric conditions can take up to several hundred years. This time can be significantly shortened (from a dozen to several hundred days) by using appropriate methods for production and utilisation. One way to accelerate the degradability of PP is to use pro-oxidant additives in its production, but without losing any mechanical properties [7]. Another approach is to create appropriate composting conditions in which dedicated bacterial strains can significantly intensify the decomposition process [8,9].

From the point of view of the filler, to increase the ecological potential of polymer composites, waste materials of various origins, for example, agricultural, industrial, or post-consumer, can be used [10,11,12,13,14,15]. For example, the addition of natural fibres (such as jute, sisal, coir, banana, hemp, kenaf, flax, cotton, bamboo, and wood) can improve sliding properties and also reduce wear in combination with traditional matrices, while offering higher biodegradability of the entire material [16,17]. Waste materials like stone fruit pits (e.g., plums and cherries) and flaxseed pomace, which typically have limited applications, can be effectively utilised as fillers in polymer composites. Cherry pits, in particular, are notable for containing substantial amounts of oil (up to 37%) that is rich in fatty acids, including linoleic, oleic, palmitic, and stearic acids. These fatty acids serve as natural lubricants, enhancing the tribological properties of the composites by reducing the coefficient of friction (COF) and increasing wear resistance. This mechanism consists of the gradual release of residual oils from the filler during friction and distribution over the contact area. This creates a self-lubrication effect, as confirmed in studies on cherry stone powder in PP composites [18]. When dealing with lubricated friction pairs, it is important to consider the naturally occurring polysaccharides found in fruit seeds. These polysaccharides may positively influence the viscosity–temperature characteristics of slightly reactive oils, such as paraffin and silicone, when they come into contact with the lubricant [19]. Among ecological fillers, the group of natural fibres is noteworthy. In terms of anti-friction properties, coir fibre’s anti-friction potential has been proven [20], along with that of palm kernel fibres [21] and Jatropha curcas L. fibres [22]. Rice ash [23] and crushed snail shells [24] have also received positive results when used as post-agricultural fillers in tribologically dedicated composites.

Most studies on the tribological properties of composites containing waste components and a polymer matrix focus on tests conducted under dry friction conditions. This focus is understandable, as these materials are typically used in applications involving low to medium loads. Research is also being conducted on potential applications of biocomposites under severe friction conditions where lubrication is required. An additional challenge here is often the limited knowledge of how composite materials interact with standard lubricants. The test results, although bounded, demonstrate promising outcomes, particularly when lubricated with oil- or water-based lubricants [25,26,27,28,29].

An important factor when using waste materials to create composites is local availability. This is closely tied to reducing the carbon footprint, which tends to increase if waste must be transported over long distances to reach the composite’s production site. This is why, for this investigation, we selected food processing waste with anti-friction properties, ensuring that the quantity of this waste was substantially high in the geographic area where it was collected and utilised, specifically in Poland. Therefore, for the purposes of our research, we chose sour cherry and plum seeds, as well as flaxseed oil, which are rich in fatty acids and polysaccharides. In 2023, Poland produced 168,650 tons of sour cherries, making it one of the largest producers in the world. The plum harvest was similarly impressive, totalling 179,720 tons in the same year [30]. In turn, 3497 tons of flaxseed oil was produced in 2023 [30]. Each of these fillers was used to produce polymer composites with low-environmental-footprint matrices, enhancing the pro-ecological nature of the manufactured materials.

## 2. Materials and Methods

### 2.1. Materials

The composition of the manufactured composites was based on two matrices: Circulen Renew C14 EP448T (a polymer belonging to the Circulen Lyondell-Basell product family based on a polypropylene copolymer [PP/CR], part of the composition of which is based on renewable raw materials such as biowaste and residual oils) and PLA (Luminy L175, Total Energies Corbion, obtained from corn flour). Selected physical properties of these materials are shown in Table 1.

Cherry and plum seed powders, along with flaxseed pomace, were used as fillers in various combinations to prepare the final composites. All fillers originated from Poland. There were two variants of filler weight content in the matrix: 15 and 25%, and two variants of filler granulation (for this article, this will be referred to as ‘d’): d < 400 μm (d-granulation, diagonal of the cuboid described on the material particle) and 400 < d < 1000 μm (for simplicity, in the article, we will use the notation d > 400 μm). The filler content of 15 wt.% was selected based on previous experimental studies, including those reported in the publications [18,19]. According to earlier findings, increasing the filler content to 15 wt.%. did not cause any deterioration in the mechanical or tribological properties. In fact, in some cases, a positive effect was observed. The 15 wt.% level was the maximum previously analysed. The 25 wt.% content was introduced in this study as a new, higher level to evaluate whether a further increase in filler content would lead to additional improvements or, conversely, trigger adverse effects such as significant deterioration in the material’s properties. All the analysed materials are listed in Table 2. The selected fractions of powdered fillers were based on sieve analysis, which revealed two major fractions above and below the 400 µm mesh size.

To create individual composite variants, the first step involved drying cherry and plum seeds in an air-circulating oven at 80 °C for 6 h (Memmert UF55 oven, Memmert GmbH + Co., Schwabach, Germany). Afterwards, the dried seeds were crushed using an ultra-centrifugal mill, with the crushing disc operating at a speed of 6000 rpm (Retsch RM200, Retsch GmbH, Haan, Germany). The materials obtained, which include powdered cherry seeds and plum seeds produced through grinding, as well as flaxseed pomace, were separated using a vibratory sieve shaker (Fritsch Analysette 3 Spartan, Fritsch, GmbH + Co., Welden, Germany). This process resulted in two fractions: d < 400 μm and d > 400 μm (400 to 1000 μm).

The PP/CR matrix composites were produced as follows: Specific amounts of PP/CR and filler (depending on the variant) were mixed in a twin-screw extruder (Zamak 16/40 EHD, Zamak Mercator Sp. z o. o., Skawina, Poland). The process involved hot mixing at barrel temperatures ranging from 170 to 193 °C, with the screw rotating at a speed of 80 rpm to ensure the formation of a homogeneous filament. The filaments were cut into pellets (with the granule size around 2 mm in diameter and 3 mm in length) using a laboratory granulator (Łukasiewicz—IMPiB, Toruń, Poland) before being injected into an injection moulding machine (Engel HLS200, Engel GmbH, Schwertberg, Austria). The temperature profile during moulding was 190 → 195 → 200 → 205 → 210 °C, and the injection pressure of the machine was 7 MPa. The mould temperature was controlled by an external cooler and set to 30 °C (Shini STM 1220, Shini Plastics Technologies Inc., New Taipei City, Taiwan). After a cooling time (30 s), 4 mm thick samples (according to ISO 527-2 [31]) were removed and kept in a dry container for at least 72 h to allow for post-production shrinkage.

The PLA matrix composites were produced as follows: Appropriate amounts of PLA and filler (depending on the variant) were subjected to a hot mixing process using a twin-screw extruder, where the barrel temperature was 180–205 °C and the screw rotation speed was 70 rpm, to mix and produce a homogeneous filament. Then, injection-moulded parts were produced in the shape of discs, each with a diameter of 50 mm and a thickness of 4 mm. This was achieved using an injection moulding machine, where the temperature of the injection unit was set in the range of 185–210 °C, and the injection pressure was maintained at 8 MPa.

The composites obtained were in the shape of paddle-shaped beams with a thickness of 4 mm, a width of 10 mm, and a length of 150 mm (for composites with a PP/CR matrix) and discs with a thickness of 4 mm and a diameter of 50 mm (for composites with a PLA matrix). From these shapes, blocks for the tribological tests with dimensions of 15 (length) × 10 (width) × 4 mm (thickness) were cut.

### 2.2. Methods

All the prepared composites were subjected to Shore D hardness tests (ISO 868). Tests were conducted using a durometer (Sauter GmbH, Freiburg im Breisgau, Germany) at a temperature of 22 ± 1 °C. For each type of sample, five measurements were taken from randomly selected samples. The mean value was calculated, and the standard deviation of the measurements was determined.

Thermal conductivity measurements of the composite materials were performed for all variants. For this, an MP-2 thermal conductivity platform (Thermtest Instruments, Hanwell, NB, Canada) equipped with a TPS-4 surface sensor was used. For each material, six measurements were taken and averaged for further analysis.

**Figure 1:**
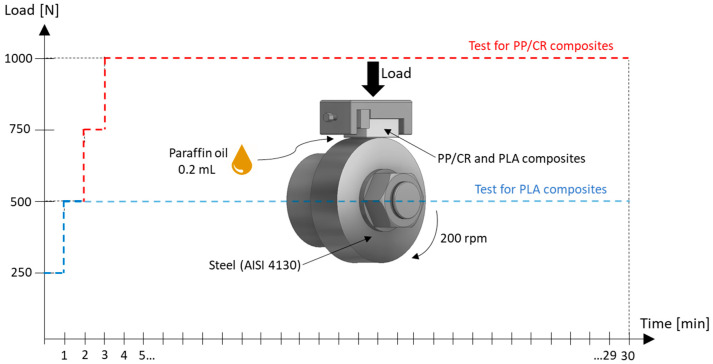
Kinetics of tribological tests.

Tribological tests to determine the COF were conducted in the ‘block-on-ring’ system (based on the Amsler type 135 tribometer, modernised and adapted for contemporary experimental requirements) under paraffin oil lubrication conditions. Paraffin oil was selected due to its low reactivity towards both steel and polymers. AISI 4130 steel rings, featuring an outer diameter of 45 mm and a width of 12 mm, were utilised as counter-samples in the tribological tests. The samples were ground to achieve an anisotropic surface topography with a roughness average (Sa) of approximately 0.5 μm. Immediately before testing, 0.2 mL of paraffin oil was applied to the cylindrical surface of the ring. The tests were conducted at a ring rotation speed of 200 rpm for 30 min, with various loads determined by the matrix material. In preliminary studies, the maximum non-seizing load for the PP/CR matrix composite was determined to be 1000 N, while for the PLA-based composite, it was found to be 500 N. The load was initially set to 250 N for each friction pair; then, every minute, this load was increased by an additional 250 N. As a result, the maximum load for PP/CR composites was reached after 3 min of testing, while for PLA composites, it was reached after just 1 min. A gradual increase in the applied load was implemented to avert rapid polymer degradation resulting from severe friction conditions triggered by the breakdown of the lubricating oil film. This approach is particularly important in the block-on-ring system, where the polymer block interfaces with a steel ring. Thanks to this, it is possible to effectively maintain the lubrication regime and eliminate the direct surface-to-surface contact that would accelerate wear. The kinetics of the tribological tests are depicted in Figure 1. During the tests, the friction torque was recorded, and based on this (and the load), the COF was determined. For each material configuration, three repetitions were performed, from which the average value and the corresponding standard deviation were calculated.

The selection of the tribological system and the testing conditions is closely related to the potential industrial applications of the composites being evaluated. A key advantage is the opportunity to use more cost-effective and environmentally friendly materials for linear bearings and low-speed sliding bearings, which are easily available from most bearing manufacturers.

SEM (FEI Quanta 250 FEG, FEI Company, Hillsboro, OR, USA) with EDS (Ametek/Edax Octane Pro, Mahwah, NJ, USA) and optical microscopy (Olympus LEXT OLS 4100, Olympus Corporation, Shinjuku City, Japan) analyses were conducted on randomly selected surfaces of both new and worn blocks following the tribological tests for each type of composite. The results from these analyses were used to identify and describe the potential wear mechanisms.

The non-polarised Raman spectra of PP, PLA, and composites with plum seed powder, cherry seed powder, and flaxseed pomace were recorded using a Renishaw inVia Raman microscope. The spectra were recorded using 785 nm wavelength laser light in the range of 100–3200 cm^−1^ with a spectral resolution better than 2 cm^−1^. The power of the laser beam focused on the sample with a ×50 objective was kept below 10 mW. The positions of the peaks were calibrated before collecting the data using a Si crystalline sample as an internal standard. The positions of bands were determined using Wire 3.4 software’s fitting package.

## 3. Results and Discussion

### 3.1. Hardness Tests

Table 3 presents the results of the hardness measurements (Shore method, test D) for all the analysed composites and base polymers. All materials containing PLA exhibited higher hardness than those made with PP/CR. Among the PP/CR materials, including both unfilled and composite variants, only the cherry seed variant of PP/CR with d < 400 μm (15 wt.%) showed lower hardness than the control sample, specifically by 1.6%. However, this difference was not statistically significant. The other composites demonstrated slightly higher hardness than the control sample, but they too were not statistically different from it. Statistically significant differences in hardness were observed only between the PP/CR composite containing 15 wt.% cherry seed filler (particle size d < 400 μm) and two composites with flaxseed pomace (particle size d > 400 μm), containing 15 wt.% and 25 wt.% filler, respectively. The hardness of the cherry seed composite was lower by 3.73% and 3.58% compared with the flaxseed variants. In turn, among the materials containing PLA, the lowest hardness value was characteristic of the PLA variant without fillers, while the highest was the PLA–plum seed variant with d > 400 μm (15 wt.%), but none of the variants were statistically different from each other.

In conclusion, it can be stated that the inclusion of fillers (sour cherry seed powder, plum seed powder, and flaxseed pomace) at the specified amounts and granulation does not significantly impact the hardness of the composites when compared to the base polymers.

### 3.2. Thermal Conductivity Measurements

Table 4 summarises the thermal conductivity values obtained for all the investigated composites and base polymers. Overall, materials based on PLA, including the pure polymer and its composites, demonstrate noticeably lower thermal conductivity compared with those based on PP/CR. This trend is most apparent when comparing the pure polymers, where PLA reaches a value of approximately 0.8 W/m·K, lower than that of PP/CR. The filler influences the thermal conductivity of the PP/CR composites, but no clear trend emerges. While the average values vary, the presence of standard deviations indicates that there is no statistically significant difference. Therefore, it can be concluded that for the PP/CR composites, the filler does not affect the thermal conductivity. For PLA and its composites, there are also no statistically significant differences between the pure polymer and the various composite variants. However, a noticeable trend can be observed: as the content and granulation of each filler increase, the thermal conductivity also tends to increase. However, these values are still (in most cases) significantly lower than for the PP/CR composites. This assumption is valid only at a constant temperature of 23 °C.

### 3.3. Tribological Tests

**Figure 2:**
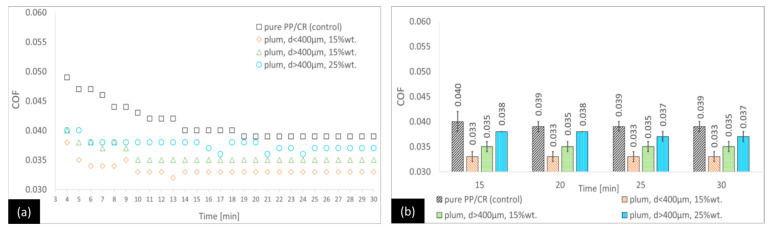
Average COF trends for the PP/CR-based composites reinforced with plum seed powder: (**a**) general trends and (**b**) selected points for the second stage of the test (with standard deviations).

**Figure 3:**
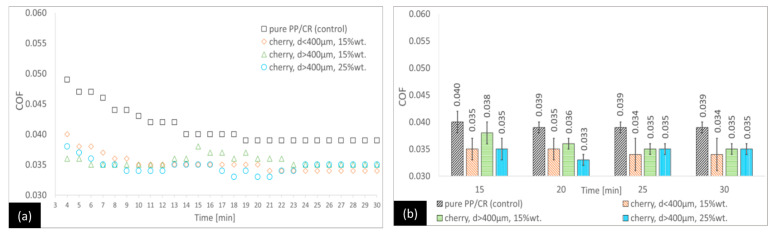
Average COF trends for the PP/CR-based composites reinforced with sour cherry seed powder: (**a**) general trends and (**b**) selected points for the second stage of the test (with standard deviations).

**Figure 4:**
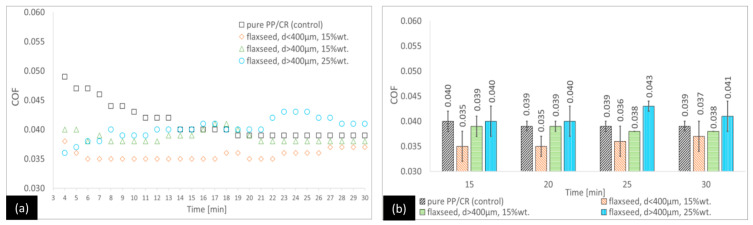
Average COF trends for the PP/CR-based composites reinforced with flaxseed pomace: (**a**) general trends and (**b**) selected points for the second stage of the test (with standard deviations).

The tribological test results will be analysed from two perspectives: the time required for the COF to stabilise and its final value after 30 min of each test. Figure 2, Figure 3 and Figure 4 show the results of the friction tests for the variants for which the matrix was PP/CR. By analysing the PP/CR–plum seed variants (Figure 2), it can be observed that the lowest COF is characterised by the PP/CR–plum seed variant with d < 400 μm (15 wt.%), followed by the PP/CR–plum seed variant with d > 400 μm (15 wt.%) and the PP/CR–plum seed variant with d > 400 μm (25 wt.%). The COF is lower than the control by 15.4%, 10.3%, and 5.1%, respectively, in the last minute of the test, although for the plum seed variant with d > 400 μm (25 wt.%), the difference is not statistically significant.

By analysing the PP/CR–cherry seed variants (Figure 3), it can be seen that at the end of the tests, all variants with filler are characterised by a statistically significant lower COF than the control, while between the individual variants (with filler), no statistical differences are observed. The differences between the reference and the variants are 12.8%, 10.3%, and 10.3%, respectively, for the PP/CR–cherry seed variant with d < 400 μm (15 wt.%), the PP/CR–cherry seed variant with d > 400 μm (15 wt.%), and the PP/CR–cherry seed variant with d > 400 μm (25 wt.%). This trend is very similar to that observed for the PP/CR–plum seed variants; however, it can be noticed that differences between the references and PP/CR variants are slightly higher in the first 14 min of tests.

For the PP/CR–flaxseed pomace variants (Figure 4), the COF values at the end of the process did not show any statistically significant differences compared with the control. However, during the first few minutes, the control sample had a COF that was significantly higher than the other variants. In the first 20 min of the tests, the only statistically significant differences observed in relation to the control occurred for the PP/CR–flaxseed pomace variant with particles smaller than 400 μm.

It is important to highlight that the PP/CR variants enriched with plum and cherry seed powder showed a significant reduction in the COF much more quickly. This stabilisation occurred on average around 9–10 min into the test. In contrast, the reference composite and the variant containing flaxseed pomace achieved COF stabilisation much later, around 19–21 min into the test.

In summary, the optimal solution was found to be composites enhanced with powders from plum and cherry seeds, characterised by a granulation size of less than 400 μm. The final COFs recorded for these materials were 0.033 and 0.034, respectively.

**Figure 5:**
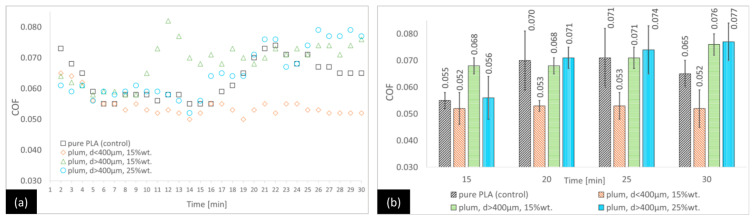
Average COF trends for the PLA-based composites reinforced with plum seed powder: (**a**) general trends and (**b**) selected points for the second stage of the test (with standard deviations).

**Figure 6:**
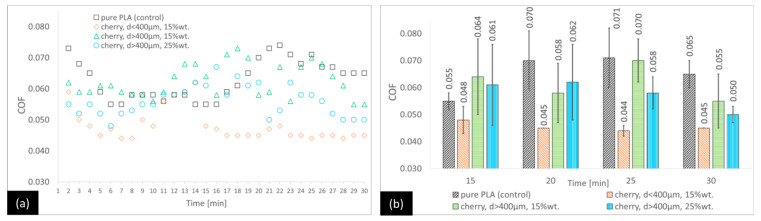
Average COF trends for the PLA-based composites reinforced with sour cherry seed powder: (**a**) general trends and (**b**) selected points for the second stage of the test (with standard deviations).

**Figure 7:**
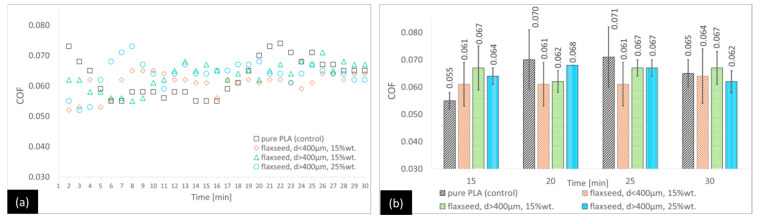
Average COF trends for the PLA-based composites reinforced with flaxseed pomace: (**a**) general trends and (**b**) selected points for the second stage of the test (with standard deviations).

Figure 5, Figure 6 and Figure 7 show the results of the friction tests for the variants in which the matrix is PLA. For the PLA–plum seed variants (Figure 5), the lowest COF at the end of the test is for the PLA–plum seed variant with d < 400 μm (15 wt.%) (24.6% lower than the control after 30 min). Higher COF values than for the control are observed for the PLA–plum seed variant with d > 400 μm (15 wt.%) (17% lower after 30 min). For the PLA–plum seed variant with d > 400 μm (25 wt.%), the difference from the control is not statistically significant. In the earlier period of the test, the COF values are variable, with the trend of the lowest COF for the PLA–plum seed variant with d < 400 μm (15 wt.%) being maintained for most of the test, starting from 8 min (between 13 and 16 min, the differences are not statistically significant).

For the PLA–cherry seed variants (Figure 6), all composites were characterised by lower COF values compared with the control at the end of the test. The lowest COF value was observed for the PLA–cherry seed variant with d < 400 μm (15 wt.%) (a 30.8% lower COF compared with the control at 30 min), followed by the PLA–cherry seed variant with d > 400 μm (25 wt.%) (a 23.0% lower COF) and the PLA–cherry seed variant with d > 400 μm (15 wt.%) (a 15.4% lower COF), with the latter variant being not statistically different from the control. The PLA–cherry seed variants exhibited specific trends in the changes in the COF over time. For options with granulation higher than 400 μm and the control sample, the COF values were variable and did not show significant statistical differences for most of the testing duration. However, the COF trend for the variant with granulation lower than 400 μm appeared to be slightly different. In this case, approximately 16 min into the test, the COF stabilised at a level that was lower (0.045–0.047) than that of the alternative composite options.

Like for the PP/CR-based components, the analysis of the PLA-flaxseed pomace variants (Figure 7) shows no statistical differences among all the options at the end of the test. In the earlier stages, the trends are variable; however, there are generally no statistically significant differences observed between the variants and the control.

In summary, for composites with a PLA matrix, the variants exhibiting the lowest COF at the end of the test are, in order from the lowest to the highest, PLA–cherry seed powder with granulation less than 400 μm (15 wt.%), PLA–cherry seed powder with granulation higher than 400 μm (25 wt.%), and PLA–plum seed powder with granulation less than 400 μm (15 wt.%). All these variants are statistically different from the control. In contrast, the COF values for all composites containing flaxseed pomace as a filler are not statistically different from the COF of the control, and it cannot be concluded if there is a positive or negative effect on friction behaviour. The results obtained for the PLA-based composites confirm the tendency visible for the PP/CR-based composites that the promising variants are primarily those with the lowest granulation (d < 400 μm) using fillers in the form of ground fruit seeds.

Based on the results of all the friction tests, it should be stated that the tribological potential of composites based on PP/CR matrices is definitely higher compared with composites based on PLA (despite more difficult friction conditions; see Section 2). This is particularly visible for fruit seed fillers, for which a lower COF is observed in most configurations than for the references and composites enriched with flaxseed pomace. It is also important to mention that the PP/CR composites containing cherry and plum seed powder fillers demonstrated greater stability, as indicated by the COF values, which stabilised after 12 min of testing. In contrast, the COF trends for the composites based on PLA varied significantly during most of the test duration.

A certain dissonance appears when analysing the mechanical properties of the base polymers (as declared by the manufacturers) (Table 1) and our hardness measurements (Table 3). It turns out that the material with the better mechanical parameters (the PLA-based materials) is subject to more intensive wear, operating under a lower load than the material with the theoretically worse initial properties (PP/CR-based materials). The lower thermal conductivity of PLA and the associated friction heat concentration in contact are probably responsible for this. This causes the rapid plasticisation of the material, which deforms intensively and is transferred to the roughness peaks of the cooperating steel surfaces.

### 3.4. SEM and Optical Microscopy Investigations

#### 3.4.1. PP/CR Composites

**Figure 8:**
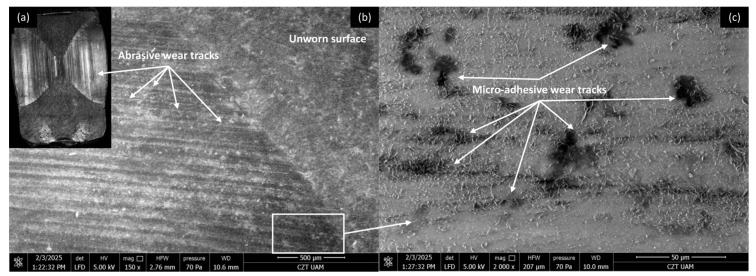
Microscopic images of the selected PP/CR polymer sample: (**a**) optical microscope without magnification; (**b**) SEM, magnification 150×; (**c**) SEM, magnification 2000×.

**Figure 9:**
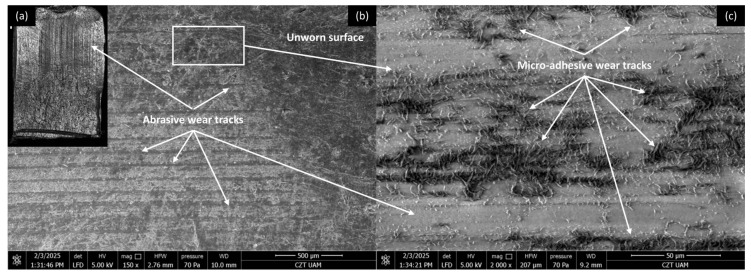
Microscopic images of the selected PP/CR-based composite sample enriched with plum seed powder (d < 400 μm, 15 wt.%): (**a**) optical microscope without magnification; (**b**) SEM, magnification 150×; (**c**) SEM, magnification 2000×.

**Figure 10:**
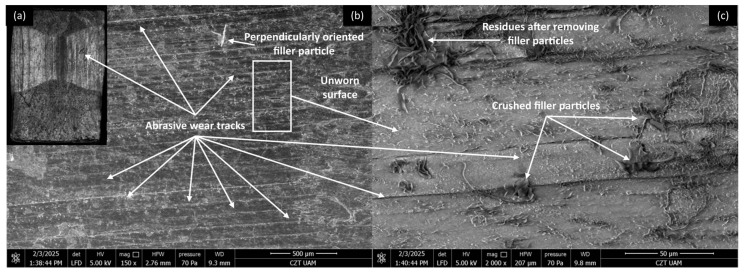
Microscopic images of the selected PP/CR-based composite sample enriched with cherry seed powder (d < 400 μm, 15 wt.%): (**a**) optical microscope without magnification; (**b**) SEM, magnification 150×; (**c**) SEM, magnification 2000×.

**Figure 11:**
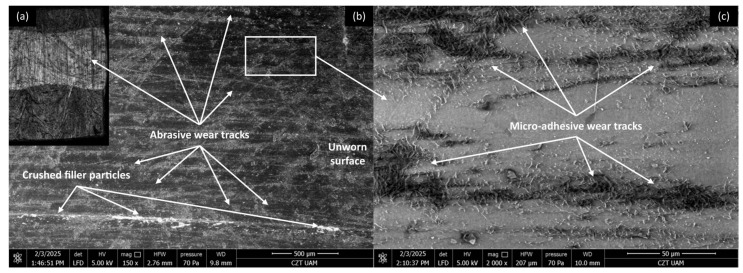
Microscopic images of the selected PP/CR-based composite sample enriched with flaxseed pomace (d < 400 μm, 15 wt.%): optical microscope without magnification (**a**); SEM, magnification 150× (**b**); SEM, magnification 2000× (**c**).

Microscopic investigations were conducted on the worn surfaces of the pure PP/CR and PLA polymers and composites containing all the analysed fillers, with the lowest granulation (<400 μm) and a content of 15 wt.% SEM pictures were meticulously captured to provide a comprehensive view, showcasing the wear track alongside a distinct fragment of the unworn surface in a single picture. Figure 8, Figure 9, Figure 10 and Figure 11 show the wear traces of the PP/CR polymer and its composites enriched with plum seed powder, cherry seed powder and flaxseed pomace, respectively.

In all cases, the dominant mechanism of wear observed is abrasive wear. Pictures taken at a higher magnification of 2000× also reveal micro-losses of material that occur due to the adhesive transfer of the polymer to the steel surface at the onset of the friction test. These losses are more prevalent in the composite materials, which is attributed to the release of filler particles as subsequent micro-layers of material are exposed during friction. Interestingly, this phenomenon can have a positive aspect. The micro-cavities formed when filler particles are removed can serve as oil pockets, creating reservoirs of lubricant. This allows for a more effective distribution of oil on the contact surface.

It is important to note that the wear tracks on the pure polymer are of a lighter colour. This observation indicates that beyond the abrasive effect, the adhesive component plays a significant role in the degradation of the polymer surface (not only at a micro level, as observed in all cases). This is related to the previously mentioned transfer of polymer material to the peaks of the steel surface roughness, which appears to occur more intensely in this case. This process is commonly referred to as ‘lumpy transfer’ [32]. The role of adhesion in the frictional destruction of composite surfaces is likely reduced due to the properties of the fillers used. Both cherry and plum pits contain fatty acids, primarily linoleic, oleic, palmitic, and stearic acids. These fatty acids are also found in small amounts in their powdered form. During friction, the destruction of filler particles allows these acids to be released and mixed with the paraffin oil used for lubrication. Because of the strong chemical affinity between the fatty acids and paraffin oil, this mixture is expected to form a homogeneous structure. Additionally, the presence of fatty acids in lubricants has been shown to enhance lubricity and improve physical adsorption on metal surfaces [18,33,34].

Cherry and plum seeds also contain polysaccharides that can aid lubrication by increasing the lubricant’s viscosity and improving its viscosity–temperature characteristics [35,36]. Their role in reducing friction and wear was confirmed in [19]. Flaxseed pomace may contain residues of linseed oil and plant mucilage polysaccharides. However, for composites enriched with this waste, no positive effect was found on the COF when interacting with steel (Figure 7).

**Figure 12:**
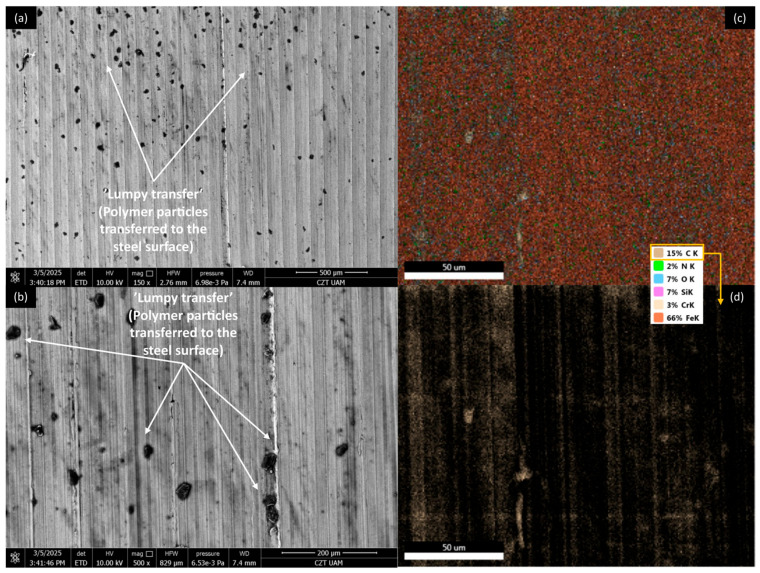
SEM images and EDS analysis of the selected AISI 4130 surface paired with the PP/CR polymer: (**a**) SEM, magnification 150×; (**b**) SEM, magnification 500×. EDS (magnification 2000×): (**c**) general distribution of selected elements; (**d**) carbon distribution.

**Figure 13:**
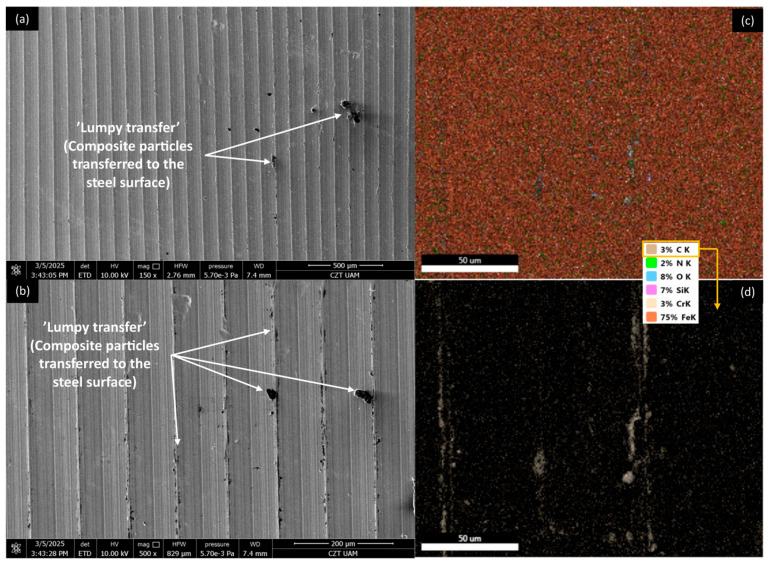
SEM images and EDS analysis of the selected AISI 4130 surface paired with the PP/CR polymer enriched with plum seed powder (d < 400 μm, 15 wt.%): (**a**) SEM, magnification 150×; (**b**) SEM, magnification 500×. EDS (magnification 2000×): (**c**) general distribution of selected elements; (**d**) carbon distribution.

**Figure 14:**
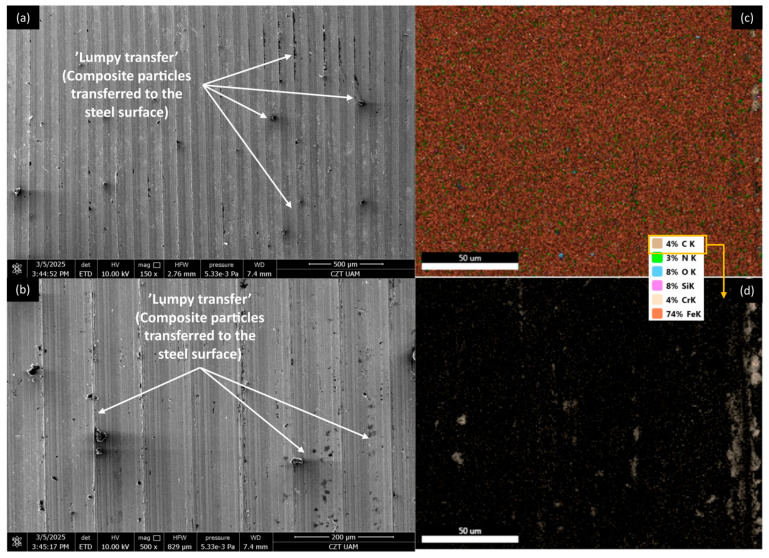
SEM images and EDS analysis of the selected AISI 4130 surface paired with the PP/CR polymer enriched with cherry seed powder (d < 400 μm, 15 wt.%): (**a**) SEM, magnification 150×; (**b**) SEM, magnification 500×. EDS (magnification 2000×): (**c**) general distribution of selected elements; (**d**) carbon distribution.

**Figure 15:**
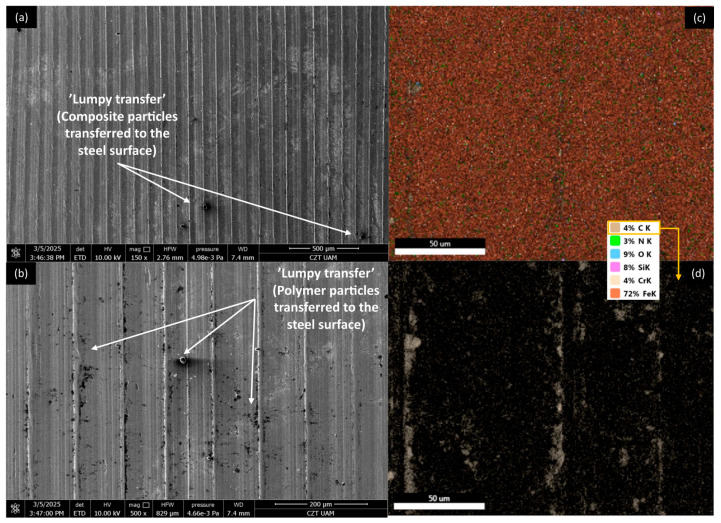
SEM images and EDS analysis of the selected AISI 4130 surface paired with the PP/CR polymer enriched with flaxseed pomace (d < 400 μm, 15 wt.%): (**a**) SEM, magnification 150×; (**b**) SEM, magnification 500×. EDS (magnification 2000×): (**c**) general distribution of selected elements; (**d**) carbon distribution.

Figure 12, Figure 13, Figure 14 and Figure 15 show AISI 4130 surfaces cooperating with the PP/CR polymer and composites enriched with plum seed powder, cherry seed powder, and flaxseed pomace, respectively. The SEM images of the wear track of the pure PP/CR polymer (Figure 12) indicate very significant friction transfer to the steel surface. The EDS analysis indicates that about 15% of the surface examined is composed of carbon (the source of which, apart from the polymer, may be the paraffin oil used for lubrication). The transfer has a typical ‘lumpy transfer’ appearance—both single lumps, non-uniformly distributed on the steel surface, and finer particles arranged along the roughness peaks in the direction of movement can be identified. For the composites enriched with plum seed (Figure 13) and cherry seed (Figure 14) powders, significantly fewer and worn particles are observed compared with the pure polymer. Only single lumps are identifiable, and the concentration along the roughness peaks is also lower (as confirmed by the C distribution, at about 3 and 4%, respectively). The surface cooperating with the flaxseed pomace-enriched composite (Figure 15) is characterised by an even smaller share of composite lumps. However, a higher concentration of worn particles along the roughness peaks is visible than for the other composites. This is visible both in the SEM images and the EDS map (in this case, the amount of C is similar to that for the plum and cherry seed powder composites).

#### 3.4.2. PLA Composites

**Figure 16:**
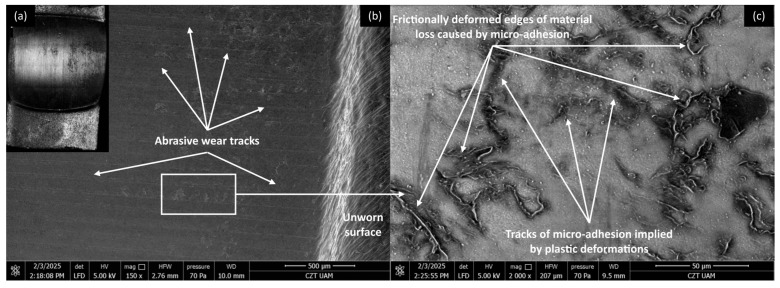
Microscopic images of the selected PLA polymer sample: (**a**) optical microscope without magnification; (**b**) SEM, magnification 150×; (**c**) SEM magnification 2000×.

**Figure 17:**
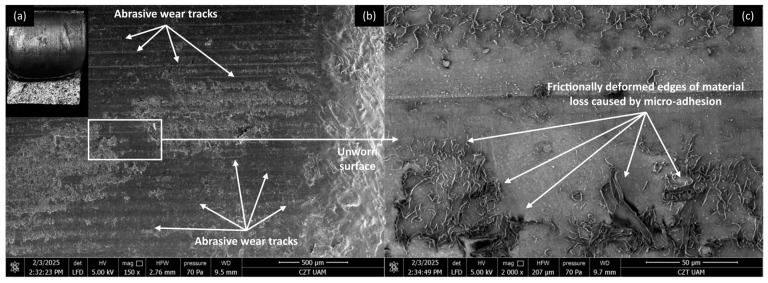
Microscopic images of the selected PLA-based composite sample enriched with plum seed powder (d < 400 μm, 15 wt.%): (**a**) optical microscope without magnification; (**b**) SEM, magnification 150×; (**c**) SEM, magnification 2000×.

**Figure 18:**
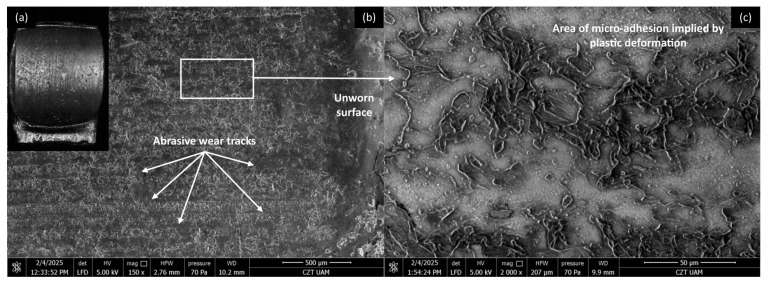
Microscopic images of the selected PLA-based composite sample enriched with cherry seed powder (d < 400 μm, 15 wt.%): (**a**) optical microscope without magnification; (**b**) SEM, magnification 150×; (**c**) SEM, magnification 2000×.

**Figure 19:**
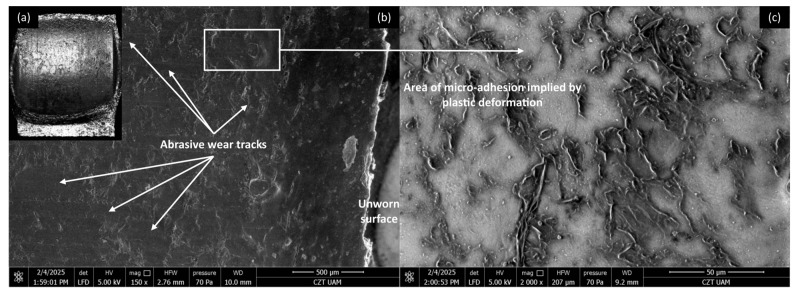
Microscopic images of the selected PLA-based composite sample enriched with flaxseed pomace (d < 400 μm, 15 wt.%): (**a**) optical microscope without magnification; (**b**) SEM, magnification 150×; (**c**) SEM, magnification 2000×.

Figure 16, Figure 17, Figure 18 and Figure 19 show the wear traces of the PLA polymer and composites enriched with plum seed powder, cherry seed powder, and flaxseed pomace, respectively. In general, the wear traces of all the PLA-based samples showed high wear (compared with the PP/CR materials) and significant influence of plastic deformation on surface destruction.

In all cases, traces of abrasive–adhesive wear are identifiable, although they differ slightly from polymers/composites based on PP/CR. The areas where the polymer or composite material transferred to the steel are the starting points for the degradation of the filler particles used. The abrasion tracks are wider and shallower compared with the PP/CR composites.

As energetically unstable locations, these are an easy path to the initiation of adhesion, resulting in the frictionally plasticised material being ‘pulled out’, which creates characteristic networks of defects. For each of the PLA-based materials, rolled edges of polymer/composite defects are visible. This plasticising effect, along with adhesive pull from the polymer surface, likely accounts for the fluctuations in the COF for PLA and its composites throughout the testing period (as shown in Figure 5, Figure 6 and Figure 7). The formation of adhesive bonds between the polymer surface and the peaks of the steel surface roughness increases resistance to motion. Once these bonds break, the resistance drops rapidly, and as a result, the COF exhibits alternating increasing and decreasing periods. This behaviour contrasts with the stabler COF trends observed for PP/CR and its composites (Figure 2, Figure 3 and Figure 4). In those cases, interactions between the polymer/composite and steel were stabler, showing a consistent decrease in COF that eventually levels off. However, the rate of stabilisation varies depending on the material variant. This suggests weaker adhesive bonding and indicates the formation of a stabler interfacial layer between the bulk polymer and the polymer deposits (‘lumpy transfer’) on the steel surface.

This mechanism is clearly linked to the previously noted poorer thermal conductivity of the PLA-based materials (Table 4). As a result, these composites exhibit pronounced macroscopic plastic deformations (Figure 16a, Figure 17a, Figure 18a and Figure 19a) and evidence of polymer/composite ‘pulling out’ (Figure 16c, Figure 17c, Figure 18c and Figure 19c).

**Figure 20:**
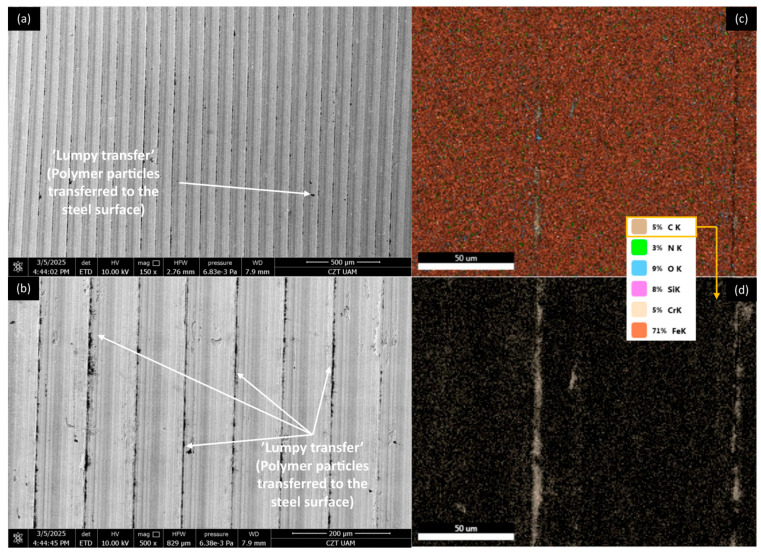
SEM images and EDS analysis of the selected AISI 4130 surface paired with the PLA polymer: (**a**) SEM, magnification 150×; (**b**) SEM, magnification 500×. EDS (magnification 2000×): (**c**) general distribution of selected elements; (**d**) carbon distribution.

**Figure 21:**
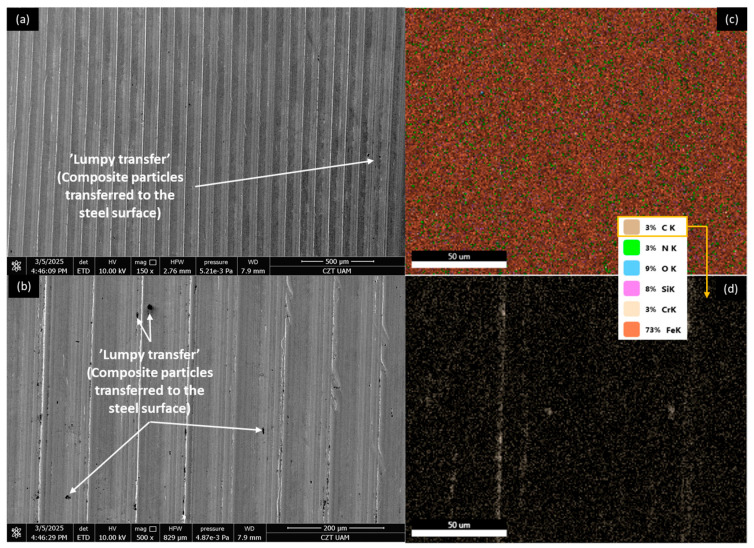
SEM images and EDS analysis of the selected AISI 4130 surface paired with the PLA polymer enriched with plum seed powder (d < 400 μm, 15 wt.%): (**a**) SEM, magnification 150×; (**b**) SEM, magnification 500×. EDS (magnification 2000×): (**c**) general distribution of selected elements; (**d**) carbon distribution.

**Figure 22:**
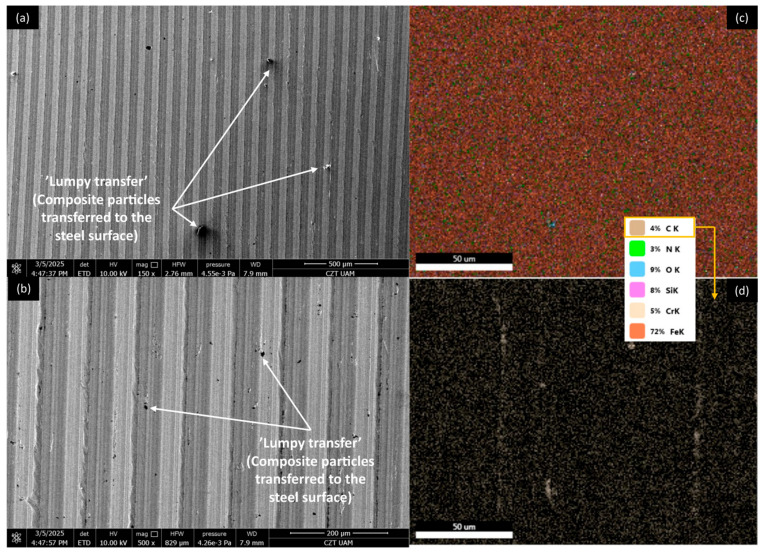
SEM images and EDS analysis of the selected AISI 4130 surface paired with the PLA polymer enriched with cherry seed powder (d < 400 μm, 15 wt.%): (**a**) SEM, magnification 150×; (**b**) SEM, magnification 500×. EDS (magnification 2000×): (**c**) general distribution of selected elements; (**d**) carbon distribution.

**Figure 23:**
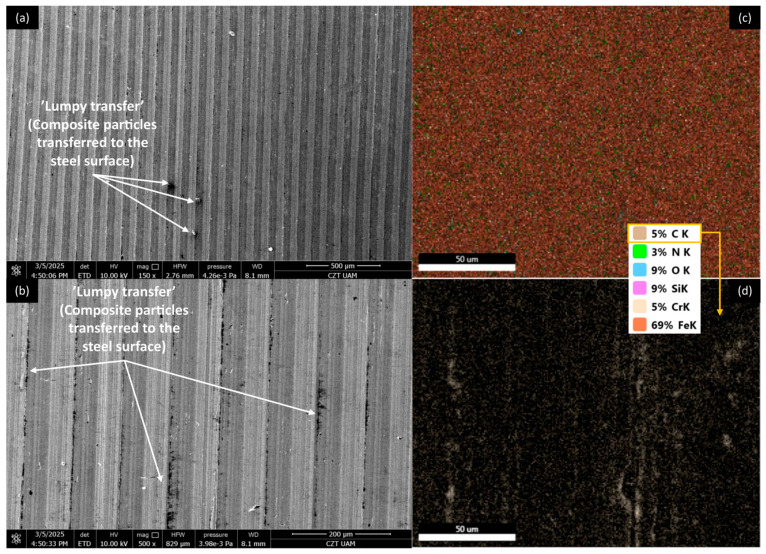
SEM images and EDS analysis of the selected AISI 4130 surface paired with the PLA polymer enriched with flaxseed pomace (d < 400 μm, 15 wt.%): (**a**) SEM, magnification 150×; (**b**) SEM, magnification 500×. EDS (magnification 2000×): (**c**) general distribution of selected elements; (**d**) carbon distribution.

Figure 20, Figure 21, Figure 22 and Figure 23 show AISI 4130 surfaces cooperating with the PLA polymer and composites enriched with plum seed powder, cherry seed powder, and flaxseed pomace, respectively. For pure PLA (Figure 20), it can be observed that the polymer transfers to the steel surface; however, its nature differs from that identified for PP/CR and its composite materials. No larger lumps of polymer were found, with transfer only occurring along the roughness peaks of the steel surface. Additionally, the amount of carbon found on the analysed surface was lower, totalling about 5%.

For AISI4130 surfaces cooperating with PLA-based composites (Figure 21, Figure 22 and Figure 23), the presence of transfer ‘lumps’ is already visible; however, they seem to be finer than for PP/CR composites. Although the analysis of the presence of carbon on these surfaces indicates similar amounts to the samples cooperating with PP/CR, the nature of the distribution is different. ‘Islands’ of carbon concentration are rarely identifiable; rather, this element is located along the roughness peaks, along the direction of movement of the rings. This form of carbon localisation indirectly confirms the significant impact of the lower thermal conductivity of PLA-based materials on thermomechanical behaviour during contact. The concentration of friction heat results in the plastic flow of the material, which is not observed as a classic ‘lumpy’ transfer. Instead, it appears as a layer of polymer or composites that conforms to the direction of movement and the roughness peaks on steel surfaces.

### 3.5. Raman Spectroscopy

**Figure 24:**
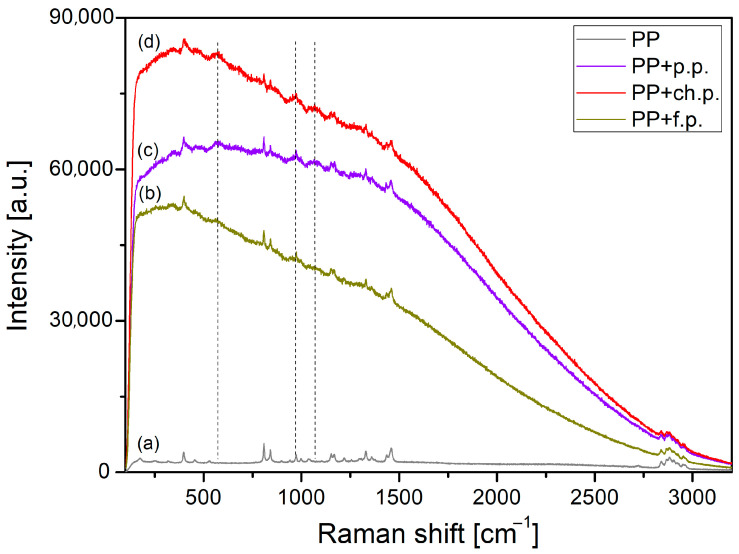
Raman spectra of (**a**) PP and composites based on PP with (**b**) flaxseed pomace, (**c**) plum seed powder, and (**d**) cherry seed powder.

**Figure 25:**
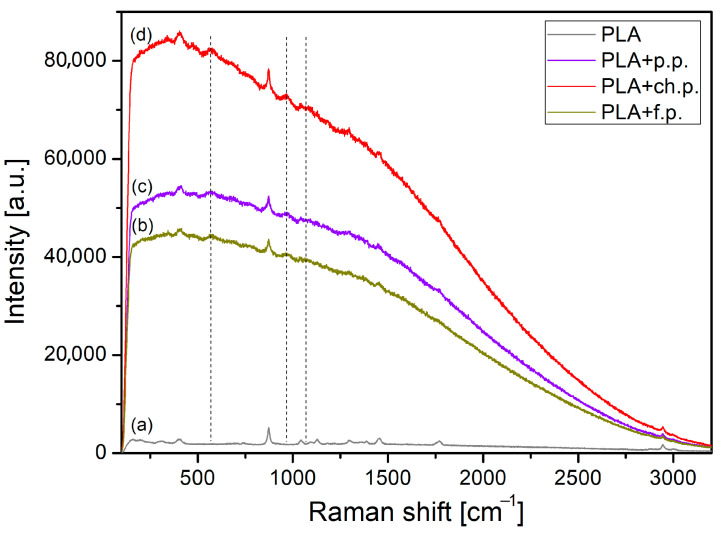
Raman spectra of (**a**) PLA and composites based on PLA with (**b**) flaxseed pomace, (**c**) plum seed powder, and (**d**) cherry seed powder.

All composites selected for Raman spectroscopy analysis correspond to the material configuration with the highest tribological potential (as indicated in Section 3.3), that is, 15 wt.% filler content and particle size d < 400 μm. The Raman spectra of pure PP and PLA and composites with plum seed powder (p.p.), cherry seed powder (ch.p.), and flaxseed pomace (f.p.) are presented in Figure 24 and Figure 25. First, it is possible to identify the characteristic spectra for both base polymers used.

The Raman spectrum of pure PP (Figure 24a) displays several prominent bands at 398, 809, 841, 1152, 1330, 1458, and 2720 cm^−1^, along with multiple bands in the 2850–3000 cm^−1^ range, confirming the polymer’s identity. The band at 398 cm^−1^ corresponds to the wagging vibration of CH_2_ and the bending of CH groups. The bands at 809 and 841 cm^−1^ are related to CH_2_ rocking and the stretching of CC skeleton and C–CH_3_ groups. The band at 1152 cm^−1^ mainly arises from the CC stretching of the backbone, C–CH_3_ stretching, CH bending, and CH_3_ rocking vibrations. The peaks at 1330 and 1458 cm^−1^ have mixed assignments: the former is due to C–H bending and CH_2_ twisting motions, while the latter corresponds to the asymmetric bending of CH_3_ and the bending of CH_2_ groups. Finally, the bands in the 2850–3000 cm^−1^ region are attributed to C–H stretching vibrations [37].

In the Raman spectrum of pure PLA (Figure 25a), a few strong bands are recorded at 305, 402, 872, 1042, 1128, 1296, 1385, 1451, and 1767 cm^−1^, and three bands in the range of 2850–3000 cm^−1^ confirm polymer identification. The assignment of the most intense bands in the spectrum is as follows: The band at 402 cm^−1^ is assigned to C–CO groups, the strongest band at 872 cm^−1^ is attributed to the stretching vibrations of C–C bonds, while the bands at 1042 and 1128 cm^−1^ are assigned to the stretching of C–CH_3_ and the rocking of CH_3_ groups, respectively. The bands in the 1290–1460 cm^−1^ range are mainly attributed to the symmetric and asymmetric deformation vibrations of CH_3_ groups. The band recorded at 1776 cm^−1^ is assigned to the stretching vibration of carbonyl groups (C=O). High wavenumber bands in the 2850–3000 cm^−1^ range are related to the symmetric and asymmetric stretching vibrations of C–H bonds in CH_3_ groups [38,39,40].

Figure 24 and Figure 25 reveal that incorporating plant-based fillers into PP/CR and PLA significantly enhances the fluorescent background. As a result, typical Raman peaks of PP and PLA become less pronounced. Nonetheless, a noteworthy feature marked by vertical dashed lines can still be observed in the spectra. Three new broad and low-intensity bands appear at about 570, 970, and 1070 cm^−1^ for the composite materials. These bands could probably be assigned to the presence of amygdalin (cyanogenic glycoside) in the cherry and plum seed powders and secoisolariciresinol diglucoside (SDG) in the flaxseed pomace as components of a polymeric composite or to polysaccharides created from amygdalin and SDG in the disintegration process. The bands are observed in the anomeric region of polysaccharides, in which the bands are rather weak and assigned to the complex skeletal vibrations of the anomeric structure of glucose [41,42,43,44,45].

A similar effect was also observed in our recently published paper [14] on polypropylene-based composites reinforced with cherry seed powder. The increase in fluorescence background in this case may be attributed to several factors: (i) the presence of amygdalin or structurally related SGDs in the plant-based fillers; (ii) the formation of polysaccharides during the degradation of amygdalin, accompanied by the generation of free radicals from cyanogenic groups or phenolic compounds; (iii) other impurities present in the fillers; and (iv) thermal oxidation occurring either during the composite manufacturing process or during tribological testing [19,46]. However, it is difficult to determine which of these factors is dominant in the studied composite materials.

Generally, the presence of polysaccharides in the contact area is a beneficial and desirable effect. This is particularly evident in lubricated friction pairs, as polysaccharides are believed to positively affect lubricant rheology [47,48] and enhance lubricity by increasing absorption capacity [35,49,50]. This was also confirmed for solutions using paraffin oil, which we used. The addition of polysaccharides increased viscosity and made it less sensitive to temperature changes [36]. In our case, we assume a similar mechanism of the influence of amygdalin and, to a lesser extent, SDG (based on tribological tests) on the friction behaviour of the analysed composites. The assumption is that the release of polysaccharides during the destruction of the composite surface by friction may cause mixing with the paraffin oil in contact, increasing local viscosity and reducing the viscosity drop generated by friction heat. For this issue, we should focus on amygdalin, since the addition of cherry and plum seed powder in each configuration with PP/CR (with PLA at the lowest concentration only) has influenced the reduction in the COF. In addition to the influence on the viscosity–temperature characteristics, attention should be paid to the antioxidant and antibacterial properties. Many natural lubricants are prone to autoxidation, leading to increased acidity, higher viscosity, and the formation of deposits. Instead of synthetic antioxidants, amygdalin could potentially inhibit the initiation and propagation of free radical reactions, thereby extending the lifespan of ‘green’ oils and greases. However, this idea should be considered speculative, as there are currently no studies that directly confirm this for use in lubricants. Nonetheless, existing research characterises the high antioxidant capacity of amygdalin (also derived from plum seeds) [51], which suggests that its potential to inhibit the oxidation of lubricants may be significant.

## 4. Conclusions

The presented studies have shown that polymer composites with fillers of agricultural waste origin can be an ecologically and functionally attractive alternative to traditional tribological materials. Based on the observations and analyses presented in these studies, the following conclusions can be stated:

- Composites made from PP/CR exhibit greater tribological potential compared with those made from PLA. The primary reason for this is the lower thermal conductivity of PLA. This results in higher friction heat within the contact zone, which leads to easier plastic deformation of the polymer/composite surface.
- For both types of composites, the optimal tribological behaviour was obtained for the lowest filler content (15 wt.%) and the lowest granulation (d < 400 μm). The exception was composites enriched with flaxseed pomace, for which the large scatter of the COF results does not allow for the statistical confirmation of this rule.
- For PP/CR composites, fillers in the form of cherry and plum seed powder for each content amount and granulation influenced the reduction in the COF concerning the pure polymer. For the PLA composites, this was statistically proven only for 15 wt.% filler content and the lowest granulation.
- Abrasive–adhesive wear characterises both groups of materials. For the PP/CR polymer and its composites, abrasive wear and the accompanying process of ‘lumpy transfer’ are predominant. The abrasion grooves on the surfaces of the polymer and composites also exhibit signs of adhesive wear and crushed filler particles. PLA and its composites exhibit significant plastic deformation, characterised by shallow and relatively wide abrasion marks. In these areas, traces of adhesive ‘pulling out’ from the materials can be observed. Additionally, ‘lumpy transfer’ is minimal, and the polymer composites tend to transfer along the roughness peaks on the cooperating steel surfaces.
- The wear traces of all composites revealed polysaccharides: amygdalin (in composites with plum and sour cherry seed powder filler) and SDG (in composites with flaxseed pomace). Their presence is related to the breakdown of filler particles caused by friction and their release at the contact area. This probably has a beneficial effect on the thermo-oxidative resistance and the viscosity–temperature characteristics of the paraffin oil used for lubrication.

## 5. Perspectives

Future research should focus on testing further variants of configurations of ecological polymer matrices and waste-derived fillers. It is also worth deepening the surface analysis to more precisely identify deposits on wear traces in the context of substances that have a beneficial effect on reducing friction and wear.

## Figures and Tables

**Table 1:** Selected physical properties of base polymers (values decelerated by manufacturers).

Property	Matrix Type	
	Circulen Renew C14 EP448T (Lyondell-Basell)	Luminy L175 (Total Energies Corbion)
Density [g/cm^3^]	0.9	1.24
Melt flow rate [g/10 min]	48 (230 °C, 2.16 kg)	8 (210 °C, 2.16 kg)
Tensile modulus [MPa]	1250	3500
Tensile strength [MPa]	27	50
Charpy impact strength [kJ/m^2^]	≤5 (23 °C)	5 (23 °C)

**Table 2:** Composite configuration variants used in the tribological tests.

Filler Type	Matrix Type	
	PP/CR (Circulen Renew C14 EP448T, Lyondell-Basell)	PLA (Total Energies Corbion)
No filler → control composite	Pure PP/CR matrix	Pure PLA matrix
Sour cherry seed powder	d < 400 μm, 15 wt.%	d < 400 μm, 15 wt.%
	d > 400 μm, 15 wt.%	d > 400 μm, 15 wt.%
	d > 400 μm, 25 wt.%	d > 400 μm, 25 wt.%
Plum seed powder	d < 400 μm, 15 wt.%	d < 400 μm, 15 wt.%
	d > 400 μm, 15 wt.%	d > 400 μm, 15 wt.%
	d > 400 μm, 25 wt.%	d > 400 μm, 25 wt.%
Flaxseed pomace	d < 400 μm, 15 wt.%	d < 400 μm, 15 wt.%
	d > 400 μm, 15 wt.%	d > 400 μm, 15 wt.%
	d > 400 μm, 25 wt.%	d > 400 μm, 25 wt.%

**Table 3:** Shore D hardness of materials (mean values and standard deviations).

Filler Type	Matrix Type	
	PP/CR (Circulen Renew C14 EP448T, Lyondell-Basell)	PLA (Luminy L175, Total Energies Corbion)
No filler → control composite	69.78 ± 1.56	82.20 ± 1.16
Sour cherry seed powder, d < 400 μm, 15 wt.%	68.67 ± 0.87	83.44 ± 1.33
Sour cherry seed powder, d > 400 μm, 15 wt.%	70.56 ± 1.51	82.75 ± 2.05
Sour cherry seed powder, d > 400 μm, 25 wt.%	70.11 ± 1.36	82.75 ± 1.58
Plum seed powder, d < 400 μm, 15 wt.%	70.0 ± 1.41	82.5 ± 1.85
Plum seed powder, d > 400 μm, 15 wt.%	70.56 ± 1.24	84.78 ± 1.48
Plum seeds powder, d > 400 μm, 25 wt.%	70.33 ± 1.12	82.22 ± 1.92
Flaxseed pomace, d < 400 μm, 15 wt.%	70.11 ± 0.93	83.5 ± 2.20
Flaxseed pomace, d > 400 μm, 15 wt.%	71.33 ± 0.87	83.88 ± 1.73
Flaxseed pomace, d > 400 μm, 25 wt.%	71.22 ± 1.39	83.0 ± 2.0

**Table 4:** Thermal conductivity of materials (mean values and standard deviations).

Filler Type	Thermal Conductivity [W/m·K]	
	PP/CR (Circulen Renew C14 EP448T, Lyondell-Basell)	PLA (Luminy L175, Total Energies Corbion)
No filler → control composite	0.277 ± 0.018	0.197 ± 0.021
Sour cherry seed powder, d < 400 μm, 15 wt.%	0.273 ± 0.030	0.221 ± 0.016
Sour cherry seed powder, d > 400 μm, 15 wt.%	0.254 ± 0.013	0.223 ± 0.021
Sour cherry seed powder, d > 400 μm, 25 wt.%	0.247 ± 0.008	0.234 ± 0.013
Plum seed powder, d < 400 μm, 15 wt.%	0.263 ± 0.017	0.207 ± 0.014
Plum seed powder, d > 400 μm, 15 wt.%	0.241 ± 0.013	0.229 ± 0.014
Plum seed powder, d > 400 μm, 25 wt.%	0.258 ± 0.011	0.231 ± 0.006
Flaxseed pomace, d < 400 μm, 15 wt.%	0.222 ± 0.012	0.208 ± 0.019
Flaxseed pomace, d > 400 μm, 15 wt.%	0.234 ± 0.018	0.216 ± 0.023
Flaxseed pomace, d > 400 μm, 25 wt.%	0.242 ± 0.025	0.238 ± 0.032

## Data Availability

The original contributions presented in this study are included in the article. Further inquiries can be directed to the corresponding author.

## Abbreviations

The following abbreviations are used in this manuscript:

PLA	polylactic acid
PP	polypropylene
COF	coefficient of friction
PP/CR	polypropylene copolymer (Circulen Renew)
SEM	scanning electron microscopy
EDS	energy-dispersive spectroscopy
